# The Toothache of Rabbi Judah ha-Nasi

**DOI:** 10.5041/RMMJ.10566

**Published:** 2026-01-28

**Authors:** Yehuda Zadik

**Affiliations:** Saligman Clinics, Faculty of Dental Medicine, The Hebrew University of Jerusalem and Hadassah Medical Center, Jerusalem, Israel

**Keywords:** Burning mouth syndrome, chronic pain, lichen planus, gingiva, necrotizing ulcerative gingivitis, oral medicine, pain, pemphigus vulgaris, postherpetic neuralgia, scurvy

## Abstract

The Talmud describes several illnesses attributed to Rabbi Judah ha-Nasi, one of the foremost leaders of the Jewish people after the destruction of the Second Temple, and the compiler of the canonical Mishnah. Among these conditions, *Tzafedina* of the oral cavity is mentioned, characterized by severe and persistent pain lasting for seven years. Although many modern scholars tend to identify this condition with scurvy, the Talmudic accounts of Rabbi Judah’s diet and lifestyle do not support a state of nutritional deficiency. Moreover, scurvy is not typically associated with oral pain. Instead, it is more plausible that the term *Tzafedina* functioned as an umbrella designation for a spectrum of oral disorders, potentially involving the teeth or gingiva. When applied to Rabbi Judah ha-Nasi, the Talmudic narrative appears to emphasize the psychological and symbolic dimensions of suffering. Thus, in addition to the possibility of organic oral pathologies with psychosomatic components that may manifest in the gingiva, the account may also correspond to chronic oral pain syndromes recognized in contemporary oral medicine.

## INTRODUCTION

Rabbi Judah ha-Nasi, also known as “Rabbi” or “Our Holy Teacher,” served as the *Nasi* (President) of the Jewish people in the late second and early third centuries CE. He is considered the greatest of the post-Temple leaders and was revered by both his contemporaries and subsequent generations.^1^ His unique combination of spiritual and political leadership inspired the saying, “From the time of Moses until Rabbi, Torah and greatness were not found together.”^2^ Around 200 CE, Rabbi Judah ha-Nasi compiled the *Mishnah*, an extraordinary codification that consolidated the oral traditions of generations. It was universally accepted in both the Land of Israel and Babylonia as the authoritative version of the Oral Torah and became a foundational text for Jewish scholarship and instruction, shaping Jewish learning from antiquity to the present day. The ensuing rabbinic discourse in the academies of both regions ultimately culminated in the compilation of the Jerusalem and Babylonian *Talmuds*, respectively.^1^

## THE TALMUDIC NARRATIVE

The Talmud recounts that Rabbi Judah ha-Nasi suffered from multiple ailments,^3^,^4^ including urinary stones (*Tzameret*), severe intestinal disease, eye disease, and chronic toothache. According to the account,^4^,^5^ Rabbi declared “Afflictions are precious” and accepted thirteen years of suffering, six years of urinary stones, and seven years of a condition called *Tzafedina*, involving dental pain. According to the Talmudic account, the magnitude of his suffering was such that, during those years, no woman miscarried in the Land of Israel, and the fields remained moist even in the absence of rain.

The Talmud attributes his suffering to a moral lesson: once, when a calf sought mercy from Rabbi as it was led to slaughter, he dismissed it saying, “Go, for you were created for this purpose,” upon which he was divinely afflicted with suffering. Years later, his recovery came after he prevented the killing of a nest of mice, quoting, “His mercy is upon all His works.”^6^ At that moment, Elijah the Prophet appeared in the guise of Rabbi Hiyya, placed his hands on the Rabbi’s teeth, and healed him.

## DISCUSSION

In modern Hebrew, the term *Tzafedina* refers to scurvy, a disease caused by vitamin C deficiency. Ascorbic acid is essential for the hydroxylation of proline to hydroxyproline in collagen synthesis, as well as for the hydroxylation of the neurotransmitter dopamine to norepinephrine. Most of the signs and symptoms of vitamin C deficiency result from its central role in collagen formation. Clinical manifestations include petechiae, subcutaneous ecchymoses, corkscrew-shaped hairs, and hyperkeratosis, with perifollicular hemorrhages being particularly characteristic. Skeletal involvement may include bone disease secondary to subperiosteal hemorrhage. Systemic symptoms include fatigue and lassitude, and neurological manifestations may present as depression and vasomotor instability.^7^

**Figure 1:**
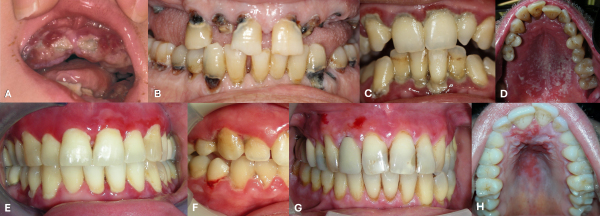
Representative Clinical Photographs Illustrating Oral Conditions Considered in the Differential Diagnosis Discussed in this Study. **A:** Gingival manifestations of scurvy in a young patient. Image courtesy of Michael Weinstein MD, Division of Paediatric Medicine, The Hospital for Sick Children, University of Toronto, Canada. **B:** Extensive carious destruction of the dentition with tooth loss, commonly accompanied by dental pain. **C:** Bacterial plaque-induced gingivitis. **D:** Oral candidiasis involving the hard palate and palatal gingiva of the maxilla. **E:** Vesiculobullous disease affecting the gingiva. **F:** Necrotizing ulcerative gingivitis. **G:** Erosive oral lichen planus involving the gingiva. **H:** Recurrent herpes simplex infection involving the hard palate and palatal gingiva of the maxilla.

In the oral cavity, gingival swelling and bleeding are common (Figure 1A). Without treatment, scurvy may be fatal, most often due to intracranial hemorrhage.^7^ The effective treatment of this condition with citrus fruits was demonstrated by the Scottish naval physician Lind in the mid-18th century, in what is considered one of the first documented controlled clinical trials.^8^

Many modern authors tend to identify the *Tzafedina* affliction attributed to Rabbi Judah ha-Nasi with scurvy in its modern medical sense.^9^–^11^ However, the Talmudic descriptions of Rabbi Judah ha-Nasi’s diet and wealth argue strongly against such a nutritional deficiency. Rabbi was known to possess orchards of balsam (*Afarsemon*),^12^ a plant identified with the biblical “oil of myrrh,” prized in antiquity for its medicinal and aromatic properties. His diet was described as rich in fruits and vegetables available throughout the year (“Neither lettuce, cucumbers, nor radishes ever ceased from his table, neither in the summer nor in the rainy season”),^13^ and some of these foods were reportedly imported from distant lands outside the Land of Israel.^1^ Furthermore, as described by Weinstein^14^ and others,^7^,^15^–^18^ oral scurvy is characterized primarily by gingival swelling and bleeding rather than pain, and certainly not by severe or chronic oral pain. It is therefore unlikely that the Talmudic term *Tzafedina* in regard to Rabbi Judah ha-Nasi referred to scurvy in its modern sense.

The disease names appearing in the Bible and the Talmud, such as *Tzara‘at*, *Se’et*, and *Sapachat*, were the accepted medico-social terms of their time and were readily understood by their audience, in accordance with the rabbinic principle that “the Torah speaks in the language of human beings.”^9^ Had these terms not been familiar, one would expect the Talmud itself to provide clarification or debate regarding their meaning. As Bar-Ilan has noted, medical terminology in the biblical and Talmudic corpus often functioned as umbrella terms, i.e. broad socio-linguistic designations encompassing a spectrum of conditions rather than discrete clinical entities in the modern sense.^19^ For example, the biblical term *Tzara‘at* did not correspond precisely to modern leprosy (as the term *Tzara‘at* refers to in modern Hebrew), but rather encompassed a wide spectrum of skin disorders and fungal infections.^19^ Similarly, the term *Tzafedina* in the Talmud likely denoted a range of oral pathologies rather than a single defined disease. Indeed, the Talmud also recounts that the Rabbi Yohanan and Abbaye, both among the greatest sages, suffered from *Tzafedina*,^20^,^21^ but there is no compelling evidence that Rabbi Judah ha-Nasi, Rabbi Yohanan, and Abbaye all suffered from the same specific illness. In fact, in the context of Rabbi Yohanan, the morbidity described is attributed to the consumption of wheat- or barley-based dishes.^21^ Therefore, the possibility of celiac disease or non-celiac gluten sensitivity cannot be excluded, conditions in which painful oral manifestations, such as ulcerations, may occur, along with additional features secondary to anemia and nutritional deficiencies.^22^,^23^ Such manifestations may historically have been subsumed under the broad and non-specific umbrella of *Tzafedina*.

An attempt to trace the underlying pathology based on its curative treatment is unproductive in this case, since there is no available information regarding the treatment of *Tzafedina* in Rabbi Judah ha-Nasi himself. Unlike his other ailments, there is no description of him seeking a cure; it is even possible that he refrained from doing so, as implied by his statement, “Afflictions are precious.” Moreover, as explained above, the remedies mentioned elsewhere in the Talmud for alleviating oral pain attributed to *Tzafedina*^20^,^21^ do not necessarily refer to the specific pathology from which Rabbi Judah ha-Nasi suffered, and therefore cannot serve to identify the nature of his condition. Even if they were related, their relevance to modern diagnostic interpretation remains doubtful, given the evolution of medical understanding, a principle known in rabbinic literature as *Hishtanut ha-teva‘im* (“the alteration of natural properties”).^11^,^24^

What, then, was the source of Rabbi Judah ha-Nasi’s oral pain? Commentators throughout the generations, as well as modern scholars, have proposed various hypotheses, suggesting that his suffering may have resulted from actual dental pain^25^,^26^ and tooth loss, gingivitis accompanied by bleeding, or oral candidiasis (Figure 1B–D).^10^,^11^,^27^ The *Mishnah Berurah* on the *Shulchan Aruch* describes *Tzafedina* as a condition “in which, when one places food in the mouth, blood flows from between the teeth,” noting that it “begins in the mouth” and that it “is dangerous”^28^, three features characteristic of vesiculobullous disease such as pemphigus vulgaris, a disease that can affect the gingiva and cause severe, persistent oral pain (Figure 1E).^29^,^30^ Shoshan as well as Dvorjetski described Rabbi Judah ha-Nasi’s oral affliction as stomatitis,^27^,^31^ a non-specific umbrella term that may correspond to various inflammatory conditions of the oral mucosa. However, except for the possibility of pemphigus, and obviously dental pain, the proposed conditions are not typically recognized as causes of such intense and enduring pain.

According to a Talmudic principle, in the narratives that the Sages chose to preserve, the moral or spiritual lesson conveyed is of primary importance, sometimes even at the expense of precise historical detail.^2^,^32^ Accordingly, attributing Rabbi Judah ha-Nasi’s suffering solely to an organic, bodily pathology may obscure the deeper ethical and spiritual message embedded in the account. Thus, the narrative conveys a profound psychological and moral dimension.^33^ As Shoshan observed, it symbolically embodies repentance and compassion—Rabbi’s pain reflecting remorse for his lack of mercy toward the calf, and his healing following an act of compassion.^31^ According to an alternative interpretation advanced by Rav Shagar, Rabbi Judah ha-Nasi did not willingly accept suffering; rather, his pain is portrayed as punitive in nature. Nevertheless, a redemptive function is attributed to this suffering, as it is understood to serve as expiation for the generation, as described above. In this context, a subsequent shift in Rabbi Judah ha-Nasi’s stance may be discerned, as evidenced by his later act of compassion toward the mice.^26^

From a modern oral medicine perspective, various conditions with a psychogenic etiologic component associated with gingival pain could correspond to the Talmudic description. The most common among them include necrotizing ulcerative gingivitis, erosive lichen planus, and recurrent herpetic stomatitis (Figure 1F–H). Of these, however, only lichen planus can be considered a chronic condition.

In addition, several chronic pain conditions are known to be associated with psychological factors. Psychosocial stress and adverse life events may serve as risk factors for herpes zoster and subsequent trigeminal postherpetic neuralgia, particularly among older adults.^34^,^35^ Burning mouth syndrome is a common neuropathic pain disorder of the oral cavity, affecting predominantly postmenopausal women, although it may also occur in younger individuals of both sexes.^36^ In most cases, the tongue is the primary site of discomfort, but pain may also involve the gingiva, either in conjunction with or independently of lingual symptoms.^37^ A key diagnostic feature is the normal appearance of the oral mucosa, with no visible lesions upon clinical examination.^27^ The pain is typically daily, lasting for at least two hours each day, often severe, and has a profound impact on the patient’s quality of life.^38^ Stress, depression, anxiety, and catastrophizing tendencies have been linked to the development and severity of the syndrome; however, no clear association has been demonstrated with specific stressful life events.^39^,^40^ The pain is notoriously difficult to manage, and after 5–7 years from onset a complete remission is reported in only about 3% of patients.^41^,^42^ Indeed, according to a Midrashic commentary from the Talmudic era,^43^ the oral affliction of Rabbi Judah ha-Nasi was not a manifestation of true physical illness but rather a form of painful distress, a description that may correspond to these modern chronic pain conditions.

## CONCLUSION

The prolonged dental pain of Rabbi Judah ha-Nasi, described in the Talmudic sources as *Tzafedina*, does not correspond to *Tzafedina* in its modern medical sense, i.e. scurvy, but rather likely reflects another condition involving the teeth or gingiva. Beyond the possibility of an organic pathology, the Talmudic narrative appears to emphasize the psychological and symbolic dimensions of suffering, which may correspond to the spectrum of chronic orofacial pain disorders recognized in contemporary oral medicine.
